# Supplementary data for the quantum chemical calculation of free radical substitution reaction mechanism of camptothecin

**DOI:** 10.1016/j.dib.2018.07.004

**Published:** 2018-07-09

**Authors:** Yujie Dai, Qingyuan Hua, Jun Ling, Chunfu Shao, Cheng Zhong, Xiuli Zhang, Yanying Hu, Liming Zhang, Yaotian Liu

**Affiliations:** Key Laboratory of Industrial Fermentation Microbiology (Tianjin University of Science & Technology), Ministry of Education, College of Bioengineering, Tianjin University of Science and Technology, No.29 of 13th Street, TEDA, Tianjin 300457, PR China

## Abstract

This data article contains the truncated view of the transition states for methyl radical attacking camptothecin at the site of 9, 10, 11, 12 and 14 in acidic conditions obtained from quantum computation of Gaussian 09 with B3LYP/6–31+G(d,p) level, also the truncated view of transition states for H abstraction by singlet O_2_ from sites of 9, 10, 11 and 12 of the intermediates of methyl combination with camptothecin and that by triplet O_2_ from site 9 of the intermediate of methyl combination with camptothecin in acidic condition are included. The corresponding parameters of reaction rate constant calculation for the formation of methyl radical from acetaldehyde, the first and second step of radical substitution of camptothecin under acidic conditions are listed. The data of the basic parameters for the computation of the total energy of the spin-projection of singlet oxygen, and the *S*^2^ values for the reactants, transition states and intermediates in the free radical substitution reaction of camptothecin are also included.

**Specifications Table**

**Table:** 

Subject area	*Chemistry*
More specific subject area	*Molecular graphics and modeling*
Type of data	*graph, figure, table*
How data was acquired	*By ChemBio3D Ultra 12.0*
Data format	*Raw*
Experimental factors	*Some transition state structures come from computation of Gaussian 09*
Data source location	*Tianjin, China.*
Data accessibility	*No*

**Value of the data**

• To facilitate the reader׳s understanding of this study.
• Extend readers׳ knowledge about the free radical reaction of camptothecin.
• To lay a foundation for further study on the mechanism of free radical substitution of natural medicines.

## 1 Data

Data provided in this article are based on computation performed applying Gaussian 09 [1] at B3LYP/6–31+G(d, p) level [2], [3] and the figures are treated using ChemBio3D Ultra 12.0 [4]. The corresponding parameters for the calculation of reaction rate constant, for the computation of the total energy of the spin-projection of singlet oxygen, and the *S*^2^ values for the reactants, transition states and intermediates in the free radical substitution reaction of camptothecin are included.

## 2 Experimental design, materials and methods

**Fig. 1:**
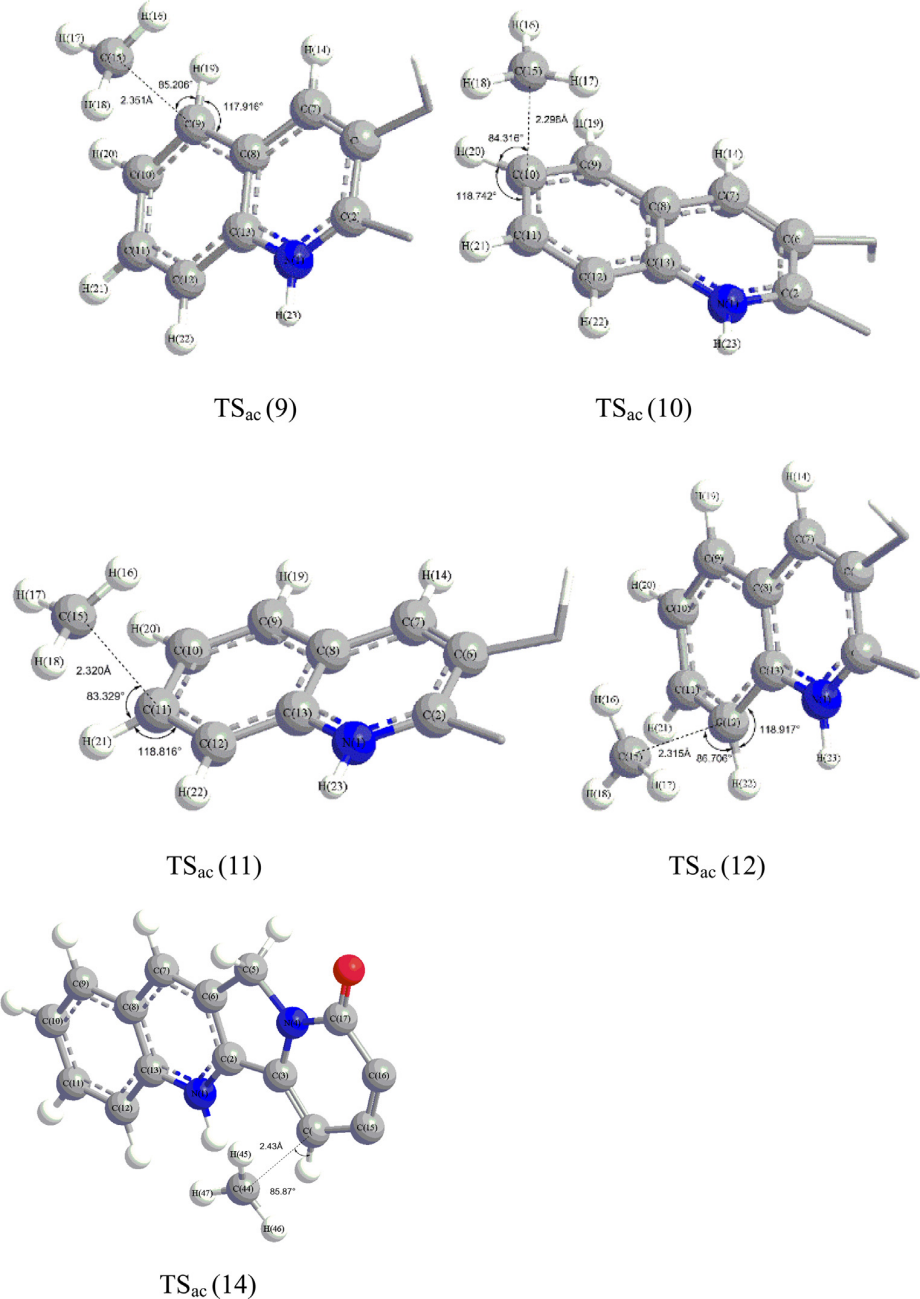
The truncated view of TS_ac_ (9), TS_ac_ (10), TS_ac_ (11), TS_ac_ (12), and TS_ac_ (14) corresponding for the transition states of methyl radical attacking camptothecin at the site of 9, 10, 11and 12 in acidic conditions respectively.

**Fig. 2:**
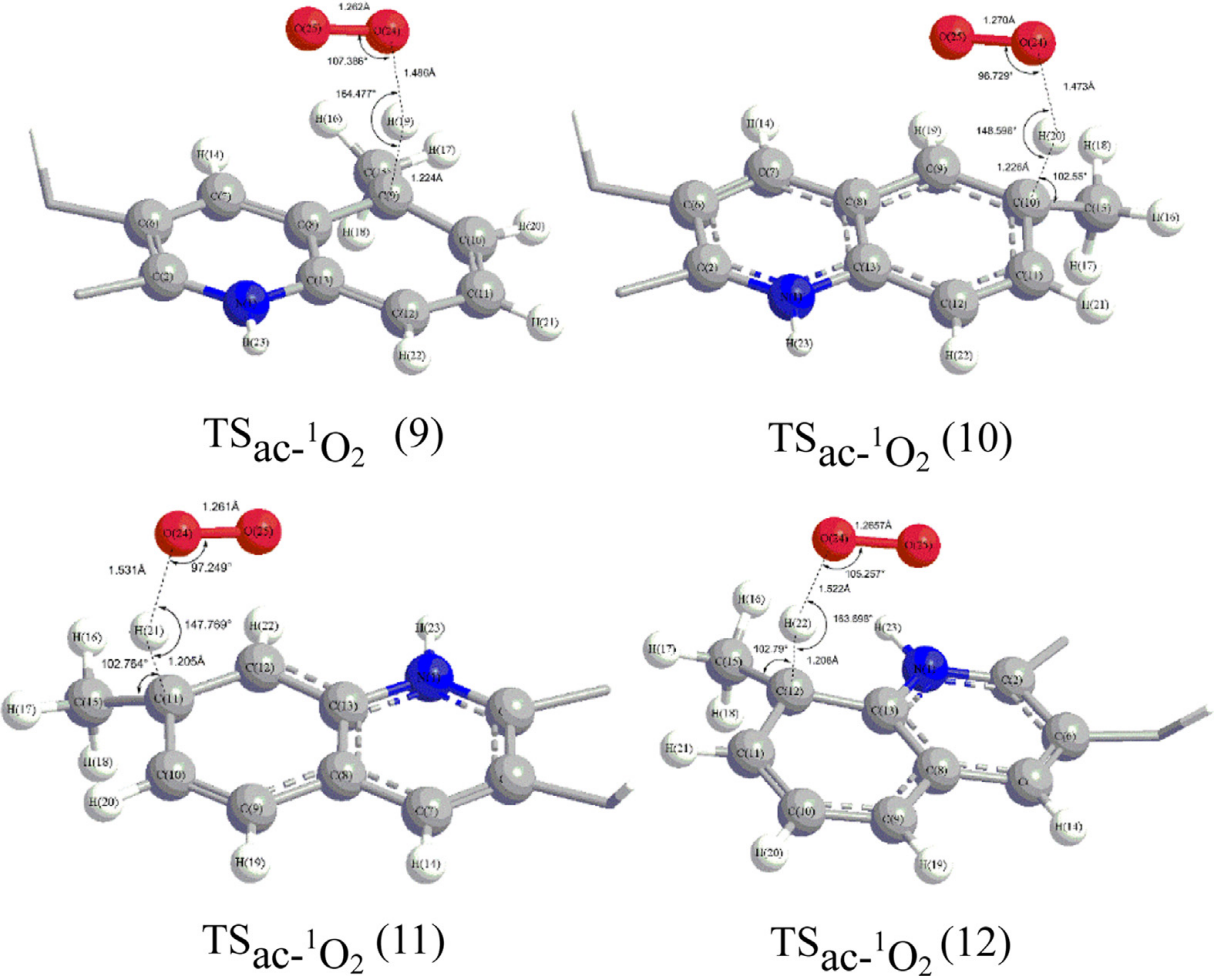
The truncated view of TS_ac-_^_1_^_O2_ (9), TS_ac-_^_1_^_O2_ (10), TS_ac-_^_1_^_O2_ (11), and TS_ac-_^_1_^_O2_ (12) corresponding for transition states of H abstraction by singlet ^1^O_2_ from sites of 9, 10, 11 and 12 of the intermediate of methyl combination with camptothecin in acidic condition respectively.

**Fig. 3:**
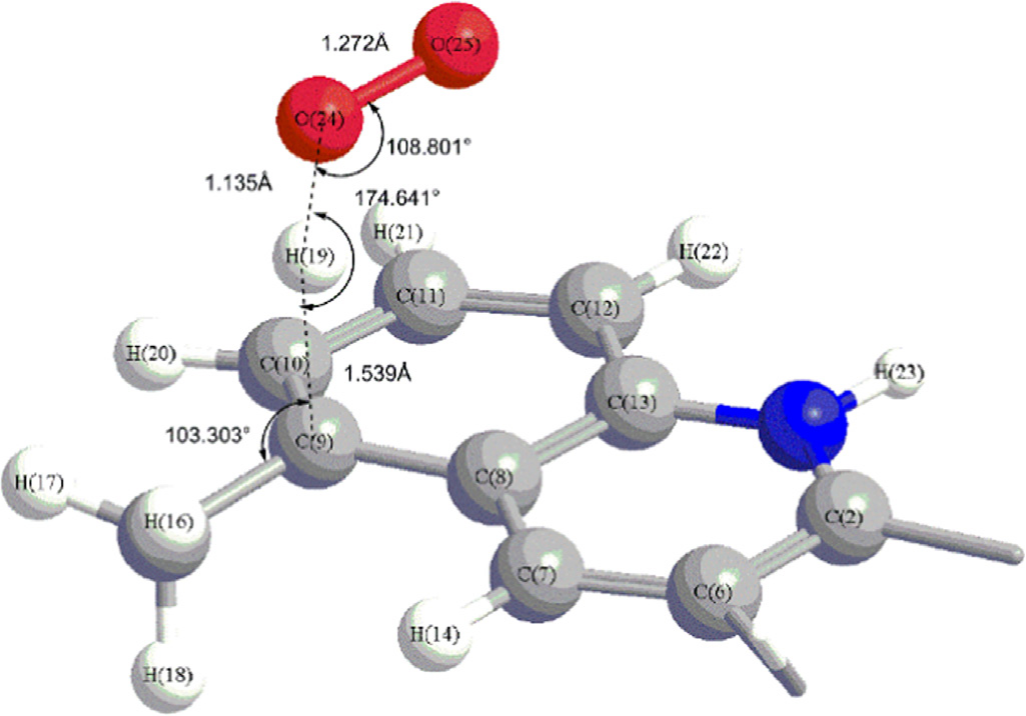
The truncated view of TS_ac-_^_3_^_O2_ (9) corresponding for the transition state of H abstraction by triplet O_2_ from site 9 of the intermediate of methyl combination with camptothecin in acidic condition.

The truncated 3D structures of the reactants, transition states (TSs) and intermediates in the reaction in Fig. 1, Fig. 2, Fig. 3 are all sketched using ChemBio3D Ultra 12.0 based on the TS optimization of the corresponding transition states with Gaussian 09 at B3LYP/6–31+G(d,p) level.

In this paper, some abbreviations were used for the convenience of description, the reactants, transition states, intermediates and products are expressed by R, TS, M and P respectively, and the Arabic numerals indicate the order. The neutral reaction conditions are denoted by n, and the acidic reaction conditions are represented by ac. The singlet O_2_ and the triplet O_2_ are denoted by ^1^O_2_ and ^3^O_2_, respectively.

*F*_*TS*_ stands for the absolute value of the frequency of the transition state. *κ* is the tunnel effect correction factor. It can be calculated by Wigner method based on the virtual frequency of the transition state. *ΔE* is the static potential threshold on the minimum energy response path (MEEP). *Q*_*A*_ and *Q*_*B*_ are the partition functions of the reactants A and B. And *Q*_*TS*_ is the partition function for the transition state.

The molecular structures and the tables are shown below:

Fig. 1 shows the truncated view of the transition states for methyl radical attacking camptothecin at the site of 9, 10, 11, 12 and 14 in acidic conditions. The truncated view of transition states for H abstraction by singlet O_2_ from sites of 9, 10, 11 and 12 of the intermediates of methyl combination with camptothecin in acidic condition is shown in Fig. 2. The structure of transition states for H abstraction by triplet O_2_ from site 9 in acidic condition is illustrated in Fig. 3.

The corresponding parameters for the calculation of reaction rate constant of the formation of methyl radical from acetaldehyde, the first and second step of radical substitution of camptothecin under acidic conditions are listed in Table 1, Table 2, Table 5 respectively. The data of the basic parameters for the computation of the total energy of the spin-projection of singlet oxygen, and the *S*^2^ values for the reactants, transition states and intermediates in the free radical substitution reaction of camptothecin are also included in Table 3, Table 4 respectively.

**Table 1:** The corresponding parameters of reaction rate constant calculation for the formation of methyl radical from acetaldehyde.

Step	*F*_*TS*_ (cm^−1^)	*κ*	*Q*_*A*_^a^	*Q*_*TS*_	*ΔE* (kcal/mol)
1	−148.84	1.022	1.24 × 10^14^	4.52 × 10^13^	3.215
2	−247.14	1.059	2.51 × 10^15^	5.47 × 10^12^	19.70

a Represents that the reactant of acetaldehyde molecule.

**Table 2:** The corresponding parameters of reaction rate constant calculation for the first step of radical substitution of camptothecin under acidic conditions.^c^ (298.15 K).

Site	*F*_*TS1*_ (cm^−1^)	*κ*	*Q*_*A1*_^a^	*Q*_*B1*_^b^	*Q*_*TS1*_	*ΔE*_*1*_ (kcal/mol)
7	−280.81	1.027	1.27 × 10^10^	4.47 × 10^22^	2.50 × 10^24^	5.42
9	−396.00	1.054	–	–	2.68 × 10^24^	8.28
10	−412.99	1.059	–	–	2.25 × 10^24^	9.16
11	−368.16	1.047	–	–	2.19 × 10^24^	8.16
12	−444.43	1.068	–	–	2.46 × 10^24^	6.88
14	−362.43	1.045	–	–	2.43 × 10^24^	7.35

a Represents that the protonated camptothecin molecule.

b Represents that the methyl radical.

c “-” represents that the value is same as above.

**Table 3:** The basic parameters for the computation of the total energy of the spin-projection of singlet oxygen.

$E^{AP}$	$E^{BS}$	$E^{HS}$	${\langle S^{2}\rangle }^{BS}$	${\langle S^{2}\rangle }^{HS}$	*α*	β
−150.324	−150.311	−150.317	2.009	1.004	2	1

**Table 4:** *S*^2^ values for the reactants, transitions and intermediates in the reaction.

Site	**Step 1**						**Step 2**		
	**Neutral conditions**			**Acidic condition**			**Acidic condition**		
	*R*_n_^a^	TS_n_^b^	*M*_n_^c^	*R*_ac_^a^	TS_ac_^b^	*M*_ac_^c^	*R*_ac-_^1^_O2_^a^	TS_ac-_^1^_O2_^b^	*P*_ac-_^1^_O2_^c^
7	–	0.7828	0.7843	–	0.7673	0.7678	0.76784	0.7662	0.7539
9	–	0.7883	0.7797	–	0.7813	0.7715	0.7715	0.7666	0.7539
10	–	0.7862	0.7867	–	0.7850	0.7844	0.7844	0.7775	0.7539
11	–	0.7835	0.7803	–	0.7738	0.7663	0.7663	0.8108	0.7539
12	–	0.7533	0.783	–	0.7883	0.7824	0.7824	0.7690	0.7539
14	–	0.7533	0.7667	–	0.7853	0.7561	–	–	–
CH_3_	0.7533	–	–	0.7533	–	–	–	–	–
^1^O_2_	–	–	–	–	–	–	1.004448	–	–

* “-” represents that the value does not exist.

**Table 5:** The corresponding parameters of reaction rate constant calculation for the second step of radical substitution of camptothecin under acidic conditions.* (298.15 K).

Site	*F*_*TS2*_ (cm^−1^)	*κ*	*Q*_*A2*_	*Q*_*B2*_	*Q*_*TS2*_	*ΔE*_*2*_ (kcal/mol)
7	−1396.24	1.054	1.02 × 10^29^	8.75 × 10^8^	1.63 × 10^25^	8.35
9	−1114.48	1.429	1.93 × 10^29^	–	1.22 × 10^6^	5.76
10	−399.56	1.055	1.65 × 10^27^	–	1.94 × 10^25^	0.98
11	−300.07	1.031	2.64 × 10^30^	–	2.15 × 10^25^	3.26
12	−922.35	1.294	3.19 × 10^28^	–	9.57 × 10^25^	6.04

^a^ represents that the intermediate produced by the first step reaction.

^b^ represents that the singlet oxygen.

^*^ “-” represents that the value is same as above.
