# Supplementary material for: Supplementary data for the quantum chemical calculation of free radical substitution reaction mechanism of camptothecin
Source: Data Brief. 2018 Jul 9;19:2305–10. doi: 10.1016/j.dib.2018.07.004 (PMC6141799; doi:10.1016/j.dib.2018.07.004)
Supplement: Supplementary file 1 — Supplementary material [file mmc1.pdf]

AUTHOR DECLARATION FOR CONFLICTS OF INTEREST

Title of paper: Supplementary data for the quantum chemical calculation of free radical substitution reaction mechanism of camptothecin.

We all authors confirm that the manuscript has been read and approved by all named authors and that there are no other persons who satisfied the criteria for authorship but are not listed. We further confirm that the order of authors listed in the manuscript has been approved by all of us.

We all authors declare that there are no known conflicts of interest associated with this publication and there has been no financial support for this work that could have influenced its outcome.

We understand that the Corresponding Author is the sole contact for the Editorial process (including Editorial Manager and direct communications with the office). He/she is responsible for communicating with the other authors about progress, submissions of revisions and final approval of proofs. We confirm that we have provided a current, correct email address which is accessible by the Corresponding Author and which has been configured to accept email from yjdai@126.com.

Corresponding author on behalf of all authors of this paper.

Printed name Yujie Dai

Signature and date Yujie Dai 2018.4.27
